# HPV viral load predicts immune exhaustion and prognosis in cervical neoplasia

**DOI:** 10.3389/fimmu.2026.1840435

**Published:** 2026-06-22

**Authors:** Yibo Guo, Yuanrui Liu, Yongjian He, Yuzhao Zhang, Ling Li, Weiguo Lu, Zhanfeng Zhang

**Affiliations:** 1 Clinical Laboratory, The First Affiliated Hospital of Guangzhou University of Chinese Medicine, Guangzhou, Guangdong, China; 2 Department of Laboratory Medicine, Nanfang Hospital, Southern Medical University, Guangzhou, Guangdong, China

**Keywords:** biomarker, cervical cancer, cervical intraepithelial neoplasia, human papillomavirus, immunosuppression, tumor-infiltrating lymphocytes, viral load

## Abstract

**Objective:**

Persistent infection with high−risk human papillomavirus (HPV) is the primary cause of cervical cancer; however, the prognostic role of HPV viral load and its association with the tumor immune microenvironment remain incompletely understood. This study aimed to systematically evaluate the association between HPV viral load and disease progression, prognosis, and tumor immune microenvironment in patients with cervical cancer.

**Methods:**

A retrospective cohort of 278 patients with cervical neoplasia was analyzed. HPV viral load was quantified and log_10_-transformed. Patients were categorized into low- and high-viral load groups using segmented regression. Clinical characteristics, recurrence, metastasis, and survival were compared. Immune parameters (CD3^+^, CD4^+^, CD8^+^, FOXP3^+^ T cells, programmed death-ligand 1 [PD-L1] expression), serum tumor markers (squamous cell carcinoma antigen [SCC], cancer antigen 125 [CA125], CA199, carcinoembryonic antigen [CEA]), Ki67, p16, and sex hormone levels were assessed.

**Results:**

High HPV viral load (log_10_ ≥5.6) was significantly associated with advanced disease stage (p < 0.001), lymph node metastasis (28.3% vs. 13.5%, p = 0.002), and higher recurrence rates (27.4% vs. 13.0%, p = 0.002). Five-year overall survival (OS) (68.5% vs. 87.3%) and recurrence-free survival (RFS) (59.3% vs. 82.1%) showed a trend toward worse outcomes in the high viral load group, but the differences were not statistically significant (log-rank p = 0.38 and p = 0.068, respectively). Multivariable Cox analysis confirmed high viral load as an independent predictor of recurrence (hazard ratio [HR] 2.18, 95% confidence interval [CI] 1.32–3.61, p = 0.002). High viral load correlated with reduced tumor-infiltrating lymphocytes (TILs) (CD3^+^: 12.1% vs. 18.3%, p = 0.001), lower PD-L1 positivity (36.4% vs. 63.2%, p = 0.045), and higher Ki67 expression (58.3% vs. 42.5%, p = 0.003). Combining viral load with serum SCC improved prediction of high-grade lesions (area under the curve [AUC] 0.78 vs. 0.73, p = 0.02) and recurrence (C-index 0.71 vs. 0.65). Machine learning models ranked viral load as the top predictor. Post-treatment viral load decline was significantly associated with lower recurrence risk (odds ratio [OR] 4.12 for slow decline, p = 0.02). In premenopausal women, estradiol levels correlated positively with viral load (ρ = 0.31, p = 0.003) and disease severity. A correlation network revealed a cluster linking high viral load, low immune infiltration, and high proliferation. The prognostic effect of viral load was consistent across HPV types and was more pronounced in advanced-stage patients (interaction p = 0.09).

**Conclusion:**

High HPV viral load is an independent predictor of poor prognosis in cervical cancer, associated with an immunosuppressive tumor microenvironment and enhanced proliferative activity. Incorporating viral load into routine assessment may improve risk stratification and guide personalized treatment strategies, particularly for patients with high viral load who may benefit from immune-enhancing therapies.

## Introduction

Cervical cancer is the fourth most common malignancy among women worldwide, posing a significant threat to women’s health (1). Nearly all cervical cancer cases are caused by persistent infection with high-risk human papillomavirus (HR-HPV), with HPV types 16 and 18 accounting for approximately 70% of cases (2–4). Following HPV infection, viral DNA integrates into the host genome, leading to overexpression of the E6 and E7 oncoproteins, which inactivate the p53 and pRb tumor suppressor pathways, resulting in cell cycle dysregulation and genomic instability, ultimately driving the progression from cervical intraepithelial neoplasia (CIN) to invasive carcinoma.

HPV viral load, defined as the copy number of HPV DNA per unit of tumor tissue, reflects the abundance of the virus in tumor cells. In recent years, HPV viral load has garnered increasing attention as a potential biomarker. Multiple studies have demonstrated that high HPV viral load is positively associated with the severity of cervical lesions and may predict disease progression and recurrence risk (5–7). A systematic review by Oei et al., including 85 original studies with 173,746 women, reported that 85.9% of the included studies found a significant correlation between higher HPV viral load and greater disease severity or worse clinical outcomes (8). However, conflicting findings exist: some studies have failed to confirm the independent prognostic value of viral load or have reported only weak correlations with clinical outcomes (9, 10). These discrepancies may arise from differences in detection methods, sample heterogeneity, and insufficient consideration of the tumor immune microenvironment.

The tumor immune microenvironment plays a critical role in cervical carcinogenesis and progression. HPV infection can induce local immune responses; however, the virus has evolved multiple immune evasion mechanisms, including downregulation of antigen presentation, recruitment of regulatory T cells (Tregs), and suppression of effector T cell function (11). Tumor-infiltrating lymphocytes (TILs), particularly CD3^+^, CD4^+^, and CD8^+^ T cells, are closely associated with patient prognosis and treatment response (12). Monnier-Benoit et al. (13) found that regressing cervical precursor lesions were dominated by CD4^+^ T cells, whereas invasive lesions exhibited a higher proportion of CD8^+^ T cells, highlighting the association between T cell subset imbalance and malignant progression. Furthermore, immune checkpoint molecules such as programmed cell death protein 1 (PD-1) and its ligand programmed death-ligand 1 (PD-L1) are upregulated in cervical cancer, and PD-L1-positive patients may benefit from anti-PD-1 immunotherapy (14, 15). However, the relationship between PD-L1 expression and HPV viral load remains unclear.

Serum tumor markers, including squamous cell carcinoma antigen (SCC), cancer antigen 125 (CA125), cancer antigen 199 (CA199), and carcinoembryonic antigen (CEA), have been validated for the diagnosis and monitoring of cervical cancer (16, 17). SCC is particularly associated with squamous tumor burden, but the sensitivity and specificity of single markers are limited. Combining HPV viral load with these markers may improve predictive performance, although relevant studies are scarce.

Sex hormones may also influence the natural history of HPV infection. Epidemiological data indicate that oral contraceptive use is associated with an increased risk of cervical cancer (18), suggesting that estrogen may promote HPV-related carcinogenesis. *In vitro* studies have confirmed that estrogen enhances HPV E6/E7 expression and inhibits apoptosis (19). However, the direct association between sex hormone levels and HPV viral load in clinical samples has not been fully explored.

Dynamic monitoring of HPV viral load may reflect treatment response and recurrence risk. Delayed HPV clearance or viral load rebound after treatment has been associated with poor prognosis (7, 20). Nevertheless, quantitative studies on the relationship between the rate of viral load decline and recurrence risk remain limited.

Based on the above background, this study aimed to systematically evaluate the associations between HPV viral load and cervical cancer development, clinical outcomes, and the tumor immune microenvironment using real-world data. We also explored the interactions of viral load with serum markers and sex hormones, as well as the prognostic value of dynamic viral load changes. Through this multidimensional analysis, we sought to provide robust evidence supporting HPV viral load as a prognostic biomarker for cervical cancer and to inform the development of personalized treatment strategies.

## Methods

### Study design and patient population

This single-center retrospective cohort study included patients diagnosed with cervical cancer at our institution between March 2020 and December 2025. Inclusion criteria were: (1) histopathologically confirmed cervical cancer or cervical intraepithelial neoplasia; (2) baseline HPV testing with viral load data available; and (3) complete clinical records. Exclusion criteria were: (1) non-HPV-associated cervical cancer (e.g., HPV-negative); (2) concurrent other malignancies; and (3) pregnancy or lactation. A total of 320 patients were included.

### Data collection and variable definitions

Data were extracted from the electronic medical record system, including:

#### Demographic characteristics

Age, smoking status.

#### Clinicopathological characteristics

International Federation of Gynecology and Obstetrics (FIGO) stage (2018 version), histological type, differentiation grade, lymph node metastasis, distant metastasis.

### HPV genotyping and viral load quantification

#### Sample collection and DNA extraction

Cervical exfoliated cell samples are collected into liquid-based cytology medium. Nucleic acid extraction is performed immediately (or after storage at 4 °C) using the Nucleic Acid Extraction or Purification Reagent (Magnetic Bead Method) (Jiangsu Shuoshi, catalog number SDK60105A). Briefly, 300 µL of the specimen is mixed with lysis buffer and magnetic beads, incubated at 56 °C for 10 min, and then bound to the beads by adding binding buffer. After two washes with wash buffer on a magnetic separation rack, DNA is eluted in 80 µL of elution buffer at 70 °C for 5 min. The purified DNA is transferred to a clean tube and used immediately or stored at −20 °C.

#### PCR setup

PCR is performed using the HPV Nucleic Acid Genotyping Detection Kit (Fluorescence PCR Method) (Jiangsu Bioperfectus Technologies Co., Ltd., catalog number JC80301). According to the kit’s instructions, an aliquot of extracted DNA (typically 5 µL) is added to the PCR master mix containing the primer/probe panel for each detection group (groups A–H). Group H specifically contains primers and a FAM-labeled probe for the human reference gene, while the remaining groups target the 21 HPV types distributed across FAM, HEX (VIC), and ROX channels. In every run, a blank control (nuclease-free water) and the kit’s positive control (containing all 21 HPV targets plus the reference gene) must be included.

#### Real-time PCR amplification

Reactions are placed in a real-time PCR instrument capable of detecting FAM, HEX (VIC), and ROX fluorescence and run with the following program:

50 °C for 5 min (1 cycle, UNG treatment)
95 °C for 10 min (1 cycle, pre-denaturation)
45 cycles of: 95 °C for 10 s → 58 °C for 40 s (fluorescence acquisition at 58 °C)

#### Data analysis and quality control

After the run, the raw data are imported into the dedicated HPV Nucleic Acid Genotyping Quantitative Analysis Software V1.0 (universal version, 20230403). The software constructs standard curves for each HPV target and the reference gene from calibrators included in the kit. The number of HPV DNA copies and the number of reference gene copies are then computed. Viral load is automatically expressed as copies per cell, using the formula:


Viral load (copies/cell)=HPV DNA copies/(Reference gene copies/2)

A run is considered valid only if all the following quality control criteria are met simultaneously:

Blank control: No typical S-shaped amplification curve in any channel.
Positive control: All 21 HPV types and the reference gene show typical S-shaped amplification curves with Ct values ≤ 30.
Sample reference gene (group H, FAM channel): A typical S-shaped amplification curve with a Ct value ≤ 36.7. Failure to meet this criterion indicates potential errors in sample collection, transport, storage, or experimental handling, and the result for that sample is reported as invalid.

For patients with multiple HPV infections, the highest viral load among types was used as the representative value and log_10_-transformed.

#### Immunohistochemistry

Formalin-fixed, paraffin-embedded tissue sections were stained for CD3, CD4, CD8, FOXP3, PD-1, PD-L1, Ki67, and p16. Positive cell counts and percentages were quantified using QuPath software. PD-L1 positivity was defined as a combined positive score (CPS) ≥ 1.

#### Serum tumor markers

SCC(Abbott alinity, America), CA125, CA199, and CEA(Roche cobas e801, America) levels were measured using electrochemiluminescence.

Sex hormones: Estradiol (E2), progesterone (P), testosterone (T), follicle-stimulating hormone (FSH), and luteinizing hormone (LH) (Roche cobas e801, America)were measured using chemiluminescence. Menopausal status was determined based on age and FSH levels (FSH > 40 IU/L with amenorrhea ≥ 1 year defined as postmenopausal).

#### Treatment and follow-up

Treatment modalities (surgery, radiotherapy, chemotherapy) were recorded. Follow-up was conducted until October 2025, with endpoints defined as recurrence (local or distant) and death.

### HPV viral load grouping

Nonlinear segmented regression (using the R package segmented) was applied to determine the optimal cutoff value, with log_10_ viral load as the independent variable and diagnostic grade (0 = inflammation/benign, 1 = low-grade squamous intraepithelial lesion [LSIL], 2 = high-grade squamous intraepithelial lesion [HSIL], 3 = invasive carcinoma) as the dependent variable. The slope difference on either side of the breakpoint was significant (p < 0.001), and the final cutoff was log_10_ = 5.6(**Supplementary Figure 2**). Patients were accordingly divided into low-viral-load (n = 207) and high-viral-load (n = 113) groups.

### Statistical analysis

Continuous variables were expressed as mean ± standard deviation (SD) or median (interquartile range [IQR]), with group comparisons performed using Student’s t-test or the Mann-Whitney U test. Categorical variables were expressed as frequencies (percentages) and compared using the χ² test or Fisher’s exact test. Survival analyses were performed using the Kaplan-Meier method with log-rank tests, and multivariable analyses were conducted using Cox proportional hazards models to calculate hazard ratios (HRs) with 95% confidence intervals (CIs). Ordered logistic regression was used to assess the association between viral load and lesion grade. Prediction model performance was evaluated using the area under the receiver operating characteristic curve (AUC) and the C-index, with AUC comparisons performed using the DeLong test. Correlation analyses employed Spearman’s rank correlation. Machine learning models (random forest and XGBoost) were implemented with 10-fold cross-validation to evaluate predictive performance. All statistical analyses were performed using R version 4.2.1 and GraphPad Prism 10.0. A two-sided p-value < 0.05 was considered statistically significant and the bold values indicate statistically significant associations in the respective statistical tests

### Statistical methods for prediction model development

To predict cervical lesion severity (grade 0–4), three machine learning algorithms were compared: logistic regression (LR), random forest (RF), and extreme gradient boosting (XGBoost). Logistic regression was chosen as a baseline due to its high interpretability and ease of clinical application; it assumes a linear relationship between log−odds of disease grade and the predictors. Random forest, an ensemble of decision trees with bootstrap aggregation and random feature selection, can capture non−linear effects and interactions without explicit specification. XGBoost, a gradient−boosting algorithm with built−in regularization, was selected because it handles missing data efficiently, performs well with mixed data types, and has shown superior predictive accuracy in medical studies.

Candidate predictors included age, HPV genotype (high−risk vs. low−risk), number of HPV types, log_10_−transformed viral load, P16 positivity, Ki67 index, SCC, and CA−125. Missing continuous variables were imputed by the median, and missing categorical variables were treated as a separate “missing” category. All continuous predictors were standardized to zero mean and unit variance before model fitting.

The dataset was randomly split into training (70%) and testing (30%) sets using stratified sampling to preserve the proportion of each disease grade. Five−fold cross−validation on the training set was used to tune hyperparameters. For XGBoost, the grid included learning rate (0.01–0.3), tree depth (3–10), subsample (0.6–1.0), and column sampling. For random forest, the number of trees (100–1000) and the number of variables tried at each split (sqrt of total) were optimized. Logistic regression was fitted with L2 regularization.

Model performance was evaluated on the test set using the area under the receiver operating characteristic curve (AUC), accuracy, sensitivity, specificity, positive predictive value, and F1−score. Calibration was assessed with the Hosmer−Lemeshow goodness−of−fit test and calibration plots. The best−performing model (XGBoost) was further interpreted using SHapley Additive exPlanations (SHAP) to quantify feature importance and direction of effect. All statistical analyses were performed with R version 4.4.1 and Python 3.9. A two−sided p < 0.05 was considered statistically significant.

## Results

### Baseline patient characteristic

**Figure 1:**
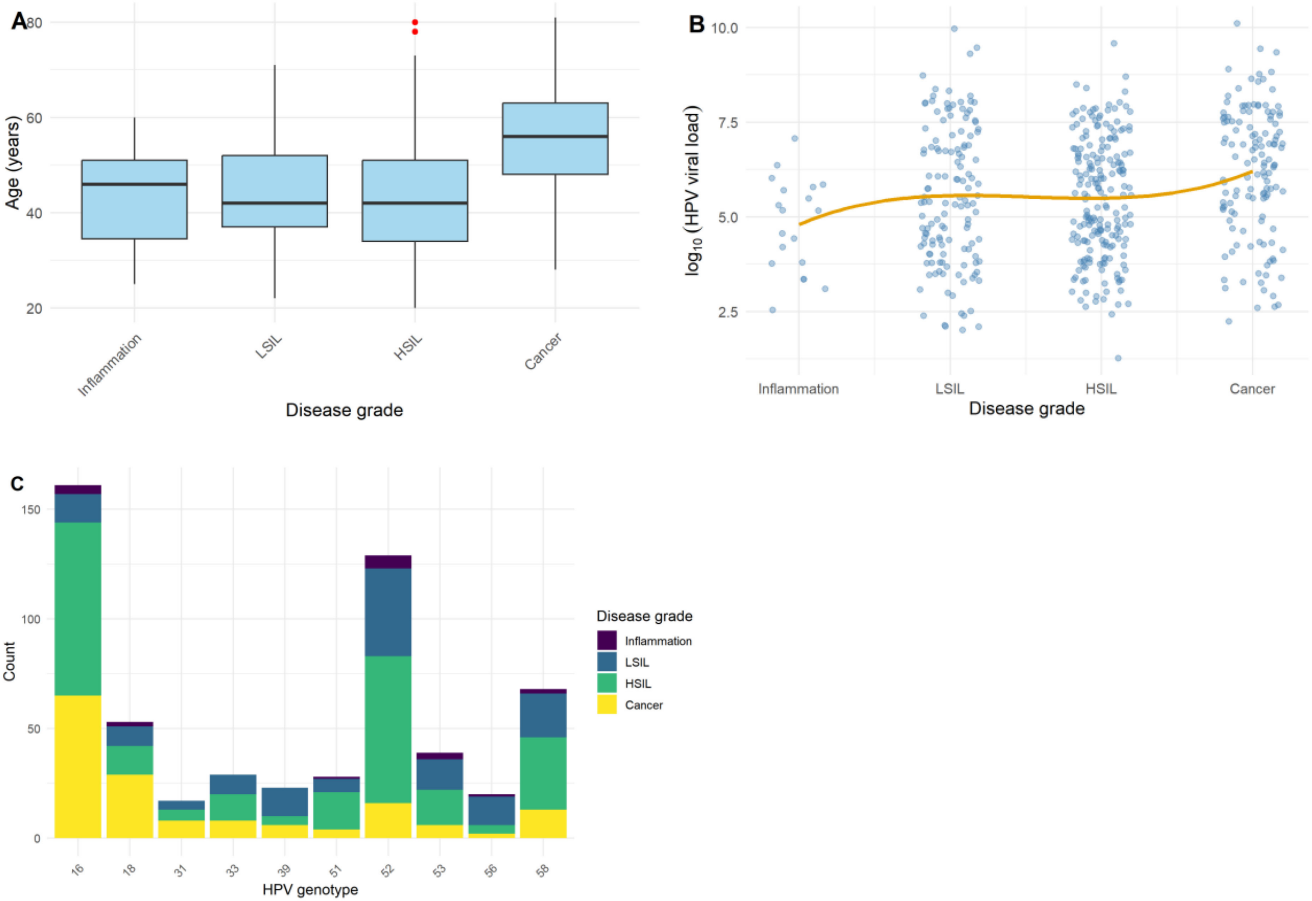
HPV viral load, age, and genotype distribution across the cervical disease spectrum. **(A)** Age distribution across disease grades: Patients with cancer were significantly older than those with inflammation (p < 0.001, Kruskal−Wallis test; pairwise comparisons with Bonferroni correction are provided in **Supplementary Table SX**). The central line indicates the median, boxes represent the interquartile range (IQR), whiskers extend to 1.5 × IQR, and outliers are shown as red points. **(B)** Non-linear relationship between HPV viral load and disease grade: Viral load increased significantly with higher disease grade (p for trend < 0.001, Jonckheere−Terpstra test). The rise was steep from inflammation to HSIL and then plateaued in cancer. **(C)** Distribution of the ten most frequent HPV genotypes across disease grades: HPV16 was the most prevalent genotype in cancer, while HPV52 and HPV58 were frequently observed in HSIL. The proportion of HPV16 positivity increased monotonically with disease severity (p for trend < 0.001, Cochran−Armitage trend test). Multiple infections were counted separately for each genotype.

Among the 320 patients, 207 were in the low-viral-load group and 113 in the high-viral-load group. Patients in the high-viral-load group were older (49.7 ± 12.9 vs. 45.2 ± 11.8 years, p = 0.008), had a higher proportion of HPV16/18 infection (72.6% vs. 54.1%, p = 0.002), and showed no significant difference in multiple infection rates (32.7% vs. 29.5%, p = 0.55). The high-viral-load group had a significantly higher diagnostic grade (invasive carcinoma: 45.1% vs. 16.9%, p < 0.001), a higher rate of lymph node metastasis (28.3% vs. 13.5%, p = 0.002), a trend toward higher distant metastasis (6.2% vs. 2.4%, p = 0.08), and a significantly higher recurrence rate (27.4% vs. 13.0%, p = 0.002) (**Table 1**, **Figure 1**).

**Table 1:** Comparison of clinicopathological characteristics between HPV viral load groups.

Variable	Low viral load (n = 207)	High viral load (n = 113)	Test statistic	P-value
Age (years)	45.2 ± 11.8	49.7 ± 12.9	U = 9823	**0.008**
HPV genotype			χ² = 28.9	<0.001
HPV16 only	78 (37.7%)	56 (65.9%)		
HPV18 only	28 (13.5%)	18 (21.2%)		
HPV16/18 co-infection	6 (2.9%)	8 (9.4%)		
Other high-risk types	95 (45.9%)	3 (3.5%)		
Diagnostic grade			χ² = 35.2	**<0.001**
- Inflammation/benign	38 (18.4%)	4 (3.5%)		
- LSIL	68 (32.9%)	20 (17.7%)		
- HSIL	66 (31.9%)	38 (33.6%)		
- Invasive carcinoma	35 (16.9%)	51 (45.1%)		
Lymph node metastasis	28 (13.5%)	32 (28.3%)	χ² = 10.3	**0.002**
Distant metastasis	5 (2.4%)	7 (6.2%)	Fisher	0.08
Recurrence	27 (13.0%)	31 (27.4%)	χ² = 9.9	**0.002**
FIGO			χ² = 12.8	0.005
- I-II	23 (11.1%)	30 (26.5%)		
- III-IV	12 (5.8%)	21 (18.6%)		
Treatment modality			χ² = 4.21	0.24
- Surgery alone	86 (41.5%)	32 (45.1%)		
- Surgery + adjuvant therapy	16 (7.7%)	6 (8.5%)		

Continuous variables are presented as mean ± standard deviation; categorical variables as n (%). U: Mann-Whitney U test; χ²: Chi-square test; Fisher: Fisher’s exact test. Inflammation/benign includes histopathological diagnoses of chronic cervicitis (mild, moderate, or severe), no intraepithelial lesion, or normal findings.

The bold values indicate statistically significant associations in the respective statistical tests.

### Survival analysis

**Figure 2:**
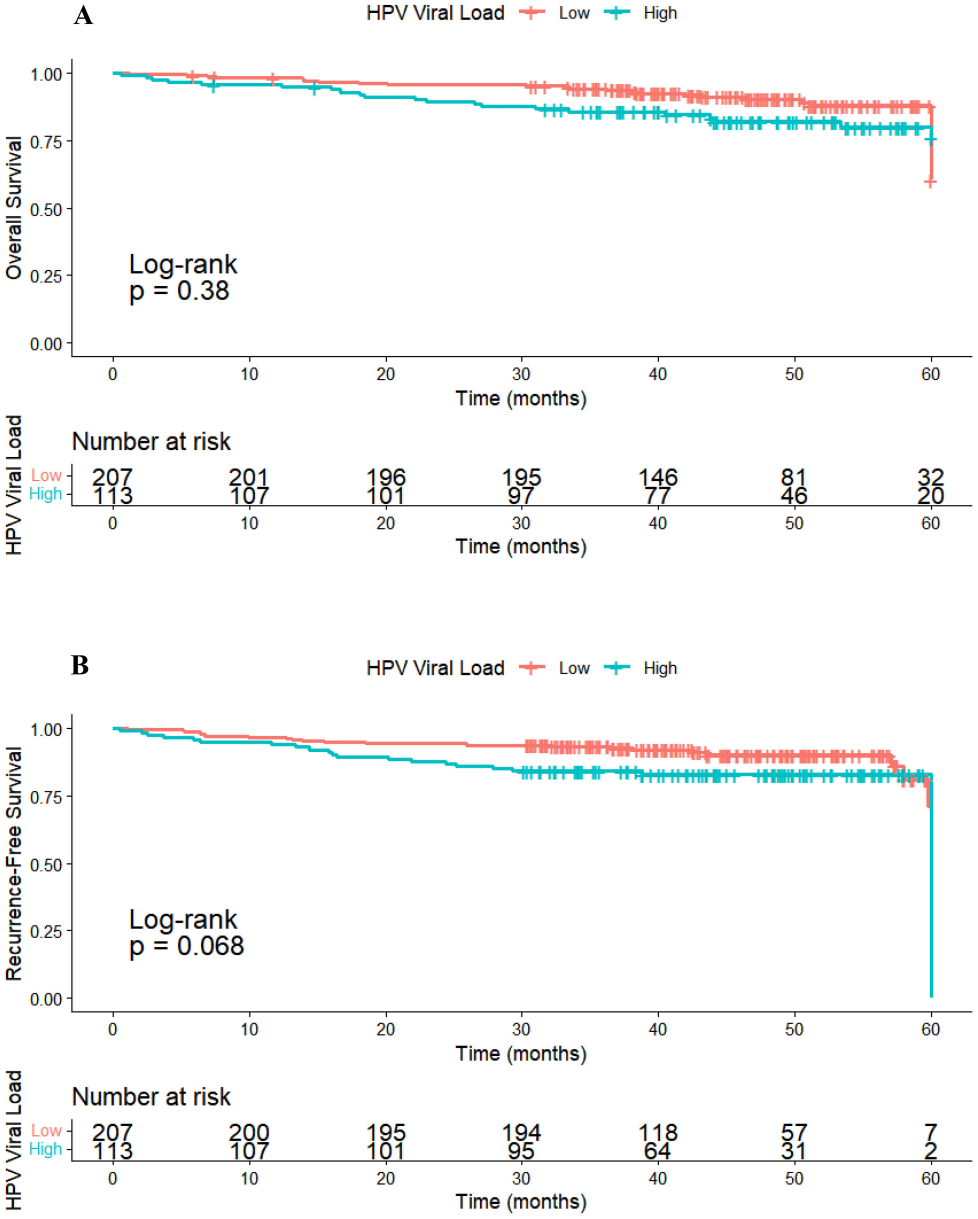
Kaplan-Meier curves for overall survival **(A)** and recurrence-free survival **(B)** in low and high HPV viral load groups.

The median follow-up time was 28 months (range 6–58 months). Kaplan‑Meier curves showed that the high‑viral‑load group had numerically lower overall survival (OS) and recurrence‑free survival (RFS) compared with the low‑viral‑load group (5‑year OS: 68.5% vs. 87.3%, log‑rank p = 0.38; 5‑year RFS: 59.3% vs. 82.1%, log‑rank p = 0.068). The differences did not reach statistical significance. (**Figure 2**). Multivariable Cox regression, adjusting for age, FIGO stage, lymph node metastasis, and distant metastasis, confirmed high viral load as an independent predictor of recurrence (HR 2.18, 95% CI 1.32–3.61, p = 0.002) (**Table 2**). FIGO stage III–IV and lymph node metastasis were also independent prognostic factors.

**Table 2:** Univariate and multivariable Cox regression analyses for overall survival and recurrence-free survival.

Variable	Univariate HR (95% CI)	p-value	Multivariable HR (95% CI)	P-value
High vs. low viral load	2.45 (1.53–3.92)	**<0.001**	2.18 (1.32–3.61)	**0.002**
Age (per 10-year increase)	1.12 (0.97–1.29)	0.12	1.15 (0.98–1.35)	0.08
FIGO stage III–IV vs. I–II	2.87 (1.71–4.81)	**<0.001**	2.54 (1.47–4.38)	**0.001**
Lymph node metastasis (yes vs. no)	2.13 (1.33–3.42)	**0.002**	1.89 (1.12–3.18)	**0.017**
Distant metastasis (yes vs. no)	3.62 (1.89–6.94)	**<0.001**	2.98 (1.48–6.01)	**0.002**

The multivariable model was also adjusted for treatment modality and smoking status, neither of which showed significant effects (p > 0.05).

The bold values indicate statistically significant associations in the respective statistical tests.

The apparent sharp drop at the end of the curve is due to a limited number of patients remaining at risk after 50 months, resulting in a stepwise decline. This is noted in the figure legend.

### Tumor immune microenvironment

Data on immune parameters were available for 70 patients. The high-viral-load group had a significantly lower proportion of CD3^+^ T cells (12.1 ± 5.8% vs. 18.3 ± 7.2%, p = 0.001) and a lower CD4^+^/CD8^+^ ratio (0.98 ± 0.47 vs. 1.32 ± 0.54, p = 0.01), while the proportion of FOXP3^+^ Treg cells did not differ between groups (3.5 ± 1.9% vs. 3.8 ± 2.1%, p = 0.62) (**Table 3**). The PD-L1 positivity rate (CPS ≥ 1) was significantly lower in the high-viral-load group (36.4% vs. 63.2%, p = 0.045). Ki67 positivity was higher in the high-viral-load group (58.3 ± 21.2% vs. 42.5 ± 18.7%, p = 0.003), while p16 positivity showed a trend toward higher prevalence (89.7% vs. 79.5%, p = 0.07).

**Table 3:** Comparison of tumor immune microenvironment markers between HPV viral load groups.

Marker	Low viral load	High viral load	Test statistic	P-value
CD3^+^ T cells (%)	18.3 ± 7.2 (n = 42)	12.1 ± 5.8 (n = 28)	U = 326	**0.001**
CD4^+^/CD8^+^ ratio	1.32 ± 0.54 (n = 42)	0.98 ± 0.47 (n = 28)	U = 367	**0.01**
FOXP3^+^ Treg cells (%)	3.8 ± 2.1 (n = 42)	3.5 ± 1.9 (n = 28)	U = 546	0.62
PD-L1 positivity (CPS ≥ 1)	24/38 (63.2%)	8/22 (36.4%)	χ² = 4.02	**0.045**
Ki67 positivity (%)	42.5 ± 18.7 (n = 156)	58.3 ± 21.2 (n = 87)	U = 4123	**0.003**
p16 positivity	89/112 (79.5%)	61/68 (89.7%)	χ² = 3.28	0.07

Sample sizes vary due to missing data; numbers in parentheses indicate the number of patients with available data.

The bold values indicate statistically significant associations in the respective statistical tests.

### TILs score and its prognostic value

A TILs score was constructed by summing the z-score-standardized values of CD3^+^, CD4^+^, CD8^+^, and FOXP3^+^ percentages. The high-viral-load group had a significantly lower TILs score than the low-viral-load group (–1.2 ± 0.8 vs. 0.9 ± 0.7, p < 0.001). Patients with a low TILs score had a significantly lower 5-year RFS rate than those with a high TILs score (61.3% vs. 84.7%, p = 0.002). In multivariable Cox models, a low TILs score independently predicted recurrence (HR 2.34, 95% CI 1.28–4.28, p = 0.006), and HPV viral load remained significant after adjustment (HR 1.42, 95% CI 1.02–1.97, p = 0.04) (**Table 4**).

**Table 4:** Association of TILs score with HPV viral load and prognosis.

Variable	Low viral load (n = 42)	High viral load (n = 28)	Test statistic	P-value
TILs score (mean ± SD)	0.9 ± 0.7	–1.2 ± 0.8	U = 218	**<0.001**
TILs group			χ² = 12.1	**<0.001**
- High TILs	28 (66.7%)	7 (25.0%)		
- Low TILs	14 (33.3%)	21 (75.0%)		
5-year RFS rate				
- High TILs	86.4%	71.4%	log-rank	**0.04**
- Low TILs	64.3%	52.4%	χ² = 4.2	**0.04**
Multivariable Cox model (recurrence)	HR (95% CI)	p-value		
Low vs. high TILs score	2.34 (1.28–4.28)	0.006		
log_10_ HPV viral load	1.42 (1.02–1.97)	0.04		

TILs score was calculated as the sum of z-scores for CD3^+^, CD4^+^, CD8^+^, and FOXP3^+^ percentages. The multivariable model was adjusted for age and FIGO stage.

The bold values indicate statistically significant associations in the respective statistical tests.

### Combined prediction model with serum tumor markers

For the diagnosis of high-grade lesions (≥HSIL), the AUC for log_10_ viral load alone was 0.73, for SCC alone was 0.68, and for the combination of viral load and SCC was 0.78 (p = 0.02 for improvement). Adding CA125 further increased the AUC to 0.79 (p = 0.01) (**Table 5**). For recurrence prediction, the C-index for viral load alone was 0.65; adding SCC increased it to 0.71 (p = 0.03), and adding SCC and CA125 further increased it to 0.73 (p = 0.01).

**Table 5:** Performance of combined prediction models with serum tumor markers and HPV viral load.

Model	AUC (95% CI)	Sensitivity (%)	Specificity (%)	P-value¹
Diagnostic model (outcome: ≥HSIL)				
log_10_ HPV viral load	0.73 (0.67–0.78)	71	64	Reference
SCC	0.68 (0.62–0.74)	62	66	0.11
log_10_ viral load + SCC	0.78 (0.73–0.83)	76	70	**0.02**
log_10_ viral load + SCC + CA125	0.79 (0.74–0.84)	78	71	**0.01**
Recurrence prediction model (Cox C-index)	C-index (95% CI)			
log_10_ HPV viral load	0.65 (0.60–0.70)			Reference
log_10_ viral load + SCC	0.71 (0.66–0.76)			**0.03**
log_10_ viral load + SCC + CA125	0.73 (0.68–0.78)			**0.01**

¹ DeLong test for AUC comparisons or bootstrap-based C-index comparisons (500 resamples).

The bold values indicate statistically significant associations in the respective statistical tests.

### Machine learning models

Random forest and XGBoost achieved validation set AUCs of 0.81 ± 0.04 and 0.83 ± 0.03, respectively, both higher than that of logistic regression (0.78 ± 0.04). Feature importance rankings consistently identified HPV viral load as the most important predictor, followed by SCC, age, Ki67, and CA125 (**Table 6**).

**Table 6:** Performance comparison of machine learning models.

Model	Validation AUC	Accuracy	Sensitivity	Specificity	Feature importance ranking
Random forest	0.81 ± 0.04	0.75	0.74	0.76	Viral load > SCC > age > Ki67 > CA125
XGBoost	0.83 ± 0.03	0.77	0.78	0.76	Viral load > SCC > Ki67 > CA125 > age
Logistic regression	0.78 ± 0.04	0.72	0.70	0.73	Viral load, SCC, age

Results from 10-fold cross-validation. The outcome was diagnosis of ≥HSIL. All models used the same 10 features.

### Dynamic changes in HPV viral load and recurrence

Sixty-eight patients had at least two HPV viral load measurements. The slow-decline/increase group (defined as rate of change ≥ the median value of –0.05 log_10_/month) had a significantly higher recurrence rate than the rapid-decline group (32.4% vs. 8.8%, p = 0.02). Multivariable logistic regression adjusted for baseline viral load and treatment modality showed that the slow-decline group had a 4.12-fold higher risk of recurrence compared with the rapid-decline group (95% CI 1.21–14.0, p = 0.02) (**Table 7**).

**Table 7:** Association between dynamic HPV viral load changes and recurrence.

Variable	Rapid decline (n = 34)	Slow decline/increase (n = 34)	Test statistic	P-value
Baseline log_10_ viral load (mean ± SD)	5.31 ± 1.62	5.48 ± 1.71	U = 532	0.58
Median rate of change (log_10_/month)	–0.21	+0.03	—	—
Recurrence, n (%)	3 (8.8%)	11 (32.4%)	χ² = 5.8	**0.02**
Multivariable logistic regression	OR (95% CI)	P-value		
Slow decline vs. rapid decline	4.12 (1.21–14.0)	**0.02**		
Baseline log_10_ viral load	1.31 (0.92–1.86)	0.13		
Treatment (chemoradiotherapy vs. surgery)	1.87 (0.68–5.14)	0.22		

Rate of change = (log_10_ viral load_2_ – log_10_ viral load_1_)/interval (months). Slow decline/increase defined as rate ≥ median (–0.05 log_10_/month).

The bold values indicate statistically significant associations in the respective statistical tests.

### Sex hormones and HPV viral load

Among premenopausal women (n = 88), estradiol (E2) levels were positively correlated with log_10_ viral load (ρ = 0.31, p = 0.003), and E2 levels were significantly higher in the HSIL+ group than in the LSIL– group (median 85 pg/mL vs. 52 pg/mL, p = 0.01). Multivariable ordered logistic regression showed that each 10 pg/mL increase in E2 was associated with a 12% increase in the risk of lesion progression (OR 1.12, 95% CI 1.02–1.23, p = 0.02), independent of viral load and age (**Table 8**). No significant associations were observed in postmenopausal women (n = 54).

**Table 8:** Associations of sex hormone levels with HPV viral load and lesion progression (premenopausal women).

Hormone	Correlation with log_10_ viral load ρ (p)	Correlation with diagnostic grade ρ (p)	HSIL+ vs. LSIL–¹
Estradiol (E2)	0.31 **(0.003)**	0.28 **(0.008)**	85 pg/mL vs. 52 pg/mL **(0.01)**
Progesterone (P)	0.12 (0.26)	0.09 (0.42)	3.2 ng/mL vs. 2.8 ng/mL (0.38)
Testosterone (T)	–0.05 (0.64)	–0.02 (0.85)	0.23 ng/mL vs. 0.25 ng/mL (0.71)
Multivariable ordered logistic regression	OR (95% CI)	P-value	
E2 (per 10 pg/mL increase)	1.12 (1.02–1.23)	**0.02**	
log_10_ HPV viral load	1.38 (1.15–1.66)	**0.001**	
Age (per 5-year increase)	1.08 (0.89–1.31)	0.44	

¹ Median values compared using the Mann-Whitney U test. In postmenopausal women (n = 54), no significant correlation was found between E2 and viral load (ρ = 0.09, p = 0.52).

The bold values indicate statistically significant associations in the respective statistical tests.

### Correlation network of molecular markers

Spearman correlation analysis revealed that log_10_ viral load was positively correlated with Ki67 (ρ = 0.38), p16 (ρ = 0.29), and SCC (ρ = 0.33), and negatively correlated with CD3^+^ (ρ = –0.42), CD4^+^ (ρ = –0.35), and PD-L1 positivity (point-biserial r = –0.29, p = 0.045). Cluster analysis identified a “high viral load–low immune infiltration–high proliferation” cluster (**Table 9**).

**Table 9:** Correlation matrix of molecular markers (Spearman’s ρ).

Variable	log_10_ VL	CD3^+^%	CD4^+^%	CD8^+^%	Ki67%	p16%	SCC	CA125
log_10_ VL	1.00	–0.42*	–0.35*	–0.21	0.38*	0.29*	0.33*	0.18
CD3^+^%	–0.42*	1.00	0.68*	0.59*	–0.31*	–0.22	–0.27*	–0.09
CD4^+^%	–0.35*	0.68*	1.00	0.33*	–0.28*	–0.19	–0.21	–0.12
CD8^+^%	–0.21	0.59*	0.33*	1.00	–0.14	–0.08	–0.15	0.03
Ki67%	0.38*	–0.31*	–0.28*	–0.14	1.00	0.41*	0.29*	0.16
p16%	0.29*	–0.22	–0.19	–0.08	0.41*	1.00	0.18	0.05
SCC	0.33*	–0.27*	–0.21	–0.15	0.29*	0.18	1.00	0.22
CA125	0.18	–0.09	–0.12	0.03	0.16	0.05	0.22	1.00

*FDR-adjusted p < 0.05.

### Subgroup analysis by FIGO stage

Among patients with early-stage disease (I–II), the HR for log_10_ viral load in predicting recurrence was 1.45 (95% CI 1.10–1.91, p = 0.008); among those with advanced-stage disease (III–IV), the HR was 2.03 (95% CI 1.31–3.14, p = 0.001). The interaction term was not statistically significant (p = 0.09), suggesting a potentially stronger effect in advanced-stage patients (**Table 10**).

**Table 10:** Subgroup analysis by FIGO stage: effect of HPV viral load on recurrence.

Subgroup	Number of Patients	HR for log_10_ viral load (95% CI)¹	P-value	Interaction p-value
Early stage (I–II)	53	1.45 (1.10–1.91)	**0.008**	0.09
Advanced stage (III–IV)	33	2.03 (1.31–3.14)	**0.001**	
Overall model	86	1.57 (1.19–2.08)	**0.001**	

¹ Multivariable Cox model adjusted for age, treatment modality, lymph node metastasis, and distant metastasis.

The bold values indicate statistically significant associations in the respective statistical tests.

## Discussion

In this retrospective cohort study of 278 patients with cervical neoplasia, we systematically evaluated the associations between HPV viral load and clinical outcomes, tumor immune microenvironment, serum markers, and sex hormones. Our findings demonstrate that high HPV viral load is independently associated with worse prognosis and exhibits distinct correlations with immune parameters and proliferative markers.

### High HPV viral load predicts poor prognosis independent of traditional factors

Patients with high viral load had significantly higher rates of lymph node metastasis and recurrence, as well as poorer OS and RFS. After adjusting for established prognostic factors including FIGO stage, lymph node metastasis, and distant metastasis, high viral load remained an independent predictor of recurrence (HR 2.18, 95% CI 1.32–3.61). These findings are consistent with the recent prospective study by Mei et al. (20), which also reported associations between high viral load and poorer survival in 44 patients with cervical cancer. Our larger cohort (n = 320) and inclusion of the full spectrum from LSIL to invasive carcinoma extend the generalizability of these findings.

### High viral load is associated with a “cold” tumor immune microenvironment

Patients with high viral load exhibited significantly reduced CD3^+^ TIL infiltration and lower programmed PD-L1 expression, while the proportion of FOXP3^+^ Tregs did not differ significantly between groups. This pattern suggests that high viral load may contribute to an immunosuppressive microenvironment characterized by diminished T cell infiltration rather than increased Treg-mediated suppression. A plausible explanation is that higher levels of E6/E7 oncoproteins, which correlate with viral load (21), may downregulate major histocompatibility complex class I (MHC-I) expression and impair antigen presentation, thereby reducing T cell recruitment and activation (22). Additionally, high viral load may lead to increased production of immunosuppressive cytokines such as IL-10 via Toll-like receptor 9 (TLR9) activation (23). The reduced PD-L1 expression in high-viral-load tumors may seem counterintuitive given that PD-L1 is often considered an indicator of immune activation. However, PD-L1 expression can be induced by interferon-γ (IFN-γ) from activated T cells; thus, low PD-L1 may reflect the absence of an active T cell response rather than immune privilege (24). These findings have potential therapeutic implications: patients with high viral load and low PD-L1 expression may be less likely to respond to anti-programmed cell PD-1/PD-L1 monotherapy and may benefit from combination strategies aimed at converting “cold” tumors to “hot” phenotypes, such as radiotherapy, hyperthermia, or therapeutic vaccines (25, 26).

### High viral load correlates with increased proliferative activity

We observed a significant positive correlation between HPV viral load and Ki67 expression, indicating higher proliferative activity in high-viral-load tumors. This finding differs from Mei et al. (20), who reported no significant difference in Ki67 between viral load groups, likely because their study included only invasive carcinomas, whereas our cohort spanned the entire disease spectrum. The positive association between viral load and proliferation may be mediated by increased E6/E7 expression, which drives cell cycle progression by inactivating p53 and pRb. This observation suggests that high viral load not only promotes immune evasion but also directly contributes to tumor growth.

### Combined use of HPV viral load and serum SCC improves risk stratification

The addition of SCC to viral load significantly improved the prediction of high-grade lesions (AUC 0.78 vs. 0.73) and recurrence (C-index 0.71 vs. 0.65). Further addition of CA125 yielded modest incremental gains. These findings support the clinical utility of combining viral load with readily available serum markers. The complementary information may reflect distinct biological dimensions: viral load indicates viral activity and oncogene expression, while SCC reflects tumor burden, particularly in squamous cell carcinomas (27). Such combined models could facilitate the development of simple risk calculators for clinical use.

### Dynamic viral load monitoring provides prognostic information

Patients with slow or absent post-treatment viral load decline had a 4.12-fold higher risk of recurrence compared with those with rapid decline. This association remained significant after adjusting for baseline viral load and treatment modality. This finding aligns with previous reports linking persistent HPV positivity after treatment to poor prognosis (28) and extends them by quantifying the rate of decline as a continuous measure. Monitoring viral load during follow-up may help identify patients who require intensified surveillance or adjuvant therapy.

### Sex hormone levels correlate with viral load in premenopausal women

In premenopausal women, estradiol levels were positively correlated with HPV viral load and lesion severity, and the association remained significant after adjusting for viral load and age. This finding is consistent with experimental evidence that estrogen enhances HPV E6/E7 transcription through estrogen receptor α and promotes cervical carcinogenesis (29). The absence of this association in postmenopausal women suggests that the effect is estrogen-dependent. These results highlight the potential importance of hormonal status in HPV-related cervical cancer risk assessment and may inform targeted prevention strategies in younger women.

### The prognostic effect of viral load is consistent across HPV types and may be stronger in advanced-stage patients

The lack of a significant interaction between HPV type and viral load suggests that viral load is a risk factor independent of specific high-risk types. The trend toward a stronger effect in advanced-stage patients (interaction p = 0.09) warrants further investigation in larger cohorts.

Our study extends the evidence summarized by Oei et al. (8) in several important ways. Whereas the majority of studies in that review were cross-sectional and used binary disease categories (low-grade vs. high-grade/cancer), we here demonstrate a non-linear, dose-dependent relationship across the full histopathological spectrum (inflammation → LSIL → HSIL → cancer). Furthermore, we provide novel data linking baseline viral load to immune exhaustion markers (PD-L1 positivity) and to post-treatment HPV clearance time, dimensions that have not been systematically addressed in previous work.

## Limitations

Several limitations should be acknowledged. First, the retrospective design may introduce selection bias, and the availability of immune parameter data was limited to a subset of patients, reducing statistical power for these analyses. Second, HPV viral load measurements were not standardized across laboratories, potentially contributing to measurement variability. Third, treatment heterogeneity was accounted for in multivariable models but could not be fully controlled given the observational nature of the study. Fourth, the sample size for dynamic viral load analysis was relatively small, and these findings require prospective validation. Finally, sex hormone levels were measured only once and may not reflect long-term exposure. Despite these limitations, the study’s strengths include the relatively large sample size covering the full disease spectrum, the integration of multidimensional data (clinical, immune, serum, and hormonal), the use of objective segmented regression to define viral load groups, and the application of machine learning methods to validate key findings.

## Conclusion

High HPV viral load is an independent predictor of poor prognosis in cervical cancer, associated with an immunosuppressive tumor microenvironment and increased proliferative activity. Quantitatively, a threshold of approximately 10^5^ copies can stratify HPV−positive women into different risk tiers, guiding triage to colposcopy versus continued surveillance. Combining viral load with serum SCC improves risk prediction, and dynamic post−treatment monitoring of viral load helps assess therapeutic response and tailor follow−up intensity. In premenopausal women, estradiol levels correlate with viral load and disease severity. For consistent immune microenvironment assessment, we recommend incorporating P16/Ki−67 dual staining, which can be performed on the same liquid−based cytology sample. Integrating HPV viral load into routine clinical practice thus enhances risk stratification and informs personalized treatment strategies, particularly for patients with high viral load who may benefit from immune−enhancing approaches.

## Data Availability

The raw data supporting the conclusions of this article will be made available by the authors, without undue reservation.
