# Supplementary material for: HPV viral load predicts immune exhaustion and prognosis in cervical neoplasia
Source: Front Immunol. 2026 Jun 22;17:1840435. doi: 10.3389/fimmu.2026.1840435 (PMC13333755; doi:10.3389/fimmu.2026.1840435)
Supplement: Supplementary file 1 [file DataSheet1.docx]

**
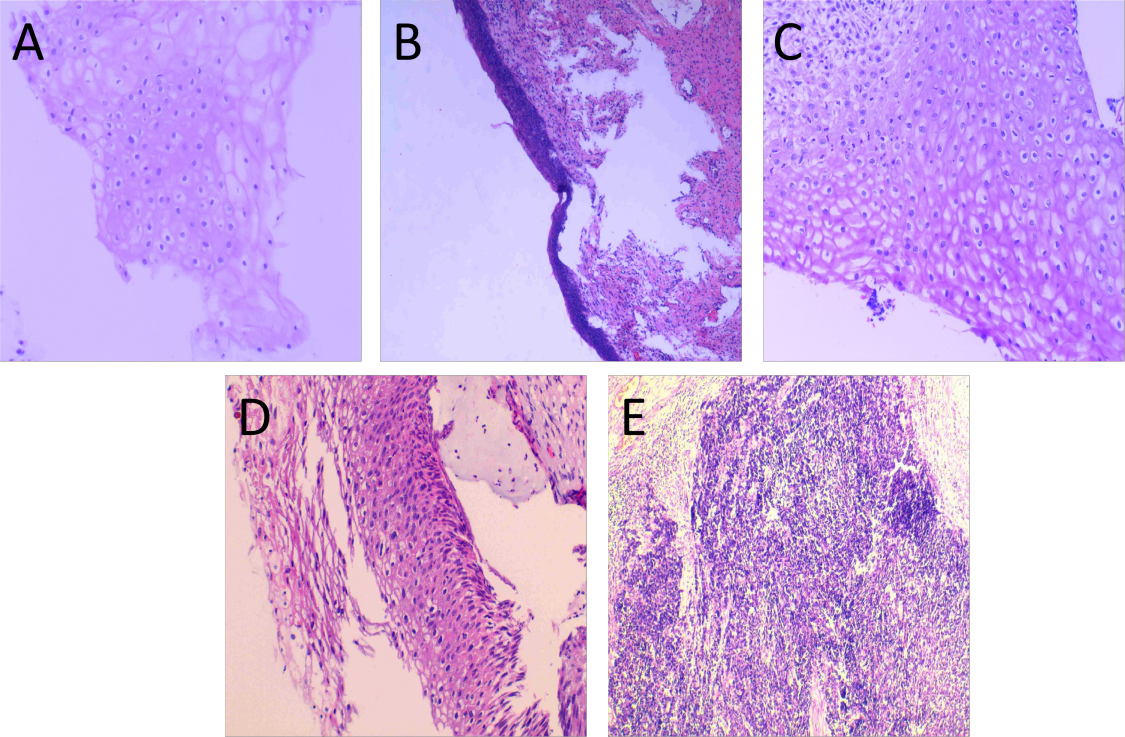
**

**Supplementary Figure S1. Histological features of cervical lesions stained with H&E(40x)**

(A) Normal/(B)Inflammatory cervical epithelium with intact basal layer and mature superficial cells.

(C)LSIL showing koilocytotic atypia (enlarged nuclei with perinuclear halos) limited to the lower one-third of the epithelium.

(D) HSIL characterized by increased nuclear-to-cytoplasmic ratio and hyperchromasia involving the lower two-thirds of the epithelium.

(E) Invasive squamous cell carcinoma characterized by irregular nests of atypical cells invading the underlying stroma.

**
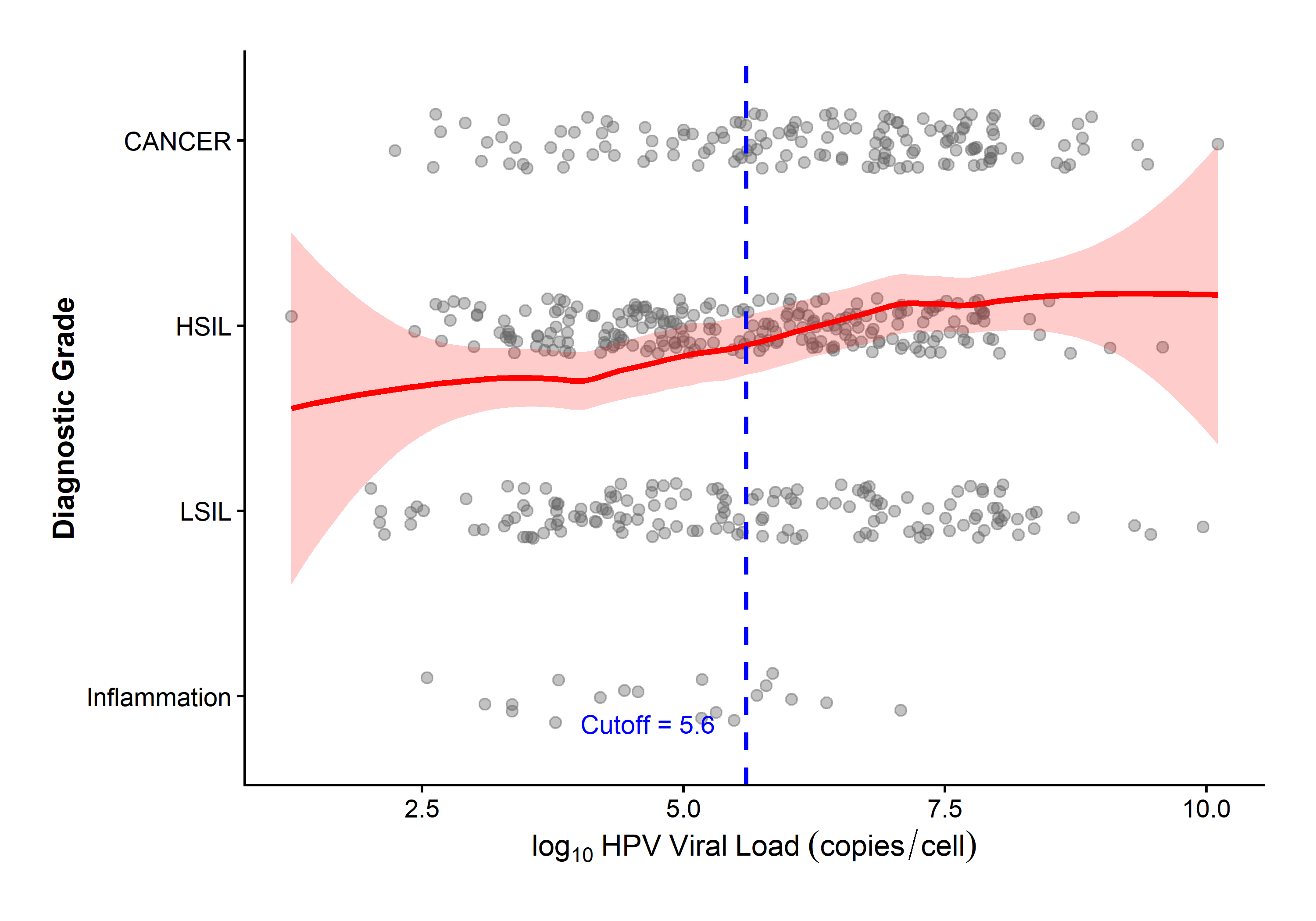
**

**Supplementary Figure S2. Nonlinear segmented regression model for determining the optimal HPV viral load cutoff.**

Scatter plot shows individual patients (N = 278) with log₁₀-transformed HPV viral load on the x-axis and diagnostic grade on the y-axis. The red line represents the piecewise linear regression fit. The vertical blue dashed line indicates the estimated breakpoint at log₁₀ = 5.6 (approximately 4.0 × 10⁵ copies).
